# *Plasmodium vivax* Malaria–associated Acute Kidney Injury, India, 2010–2011

**DOI:** 10.3201/eid1805.111442

**Published:** 2012-05

**Authors:** Vivek B. Kute, Hargovind L. Trivedi, Aruna V. Vanikar, Pankaj R. Shah, Manoj R. Gumber, Himanshu V. Patel, Jitendra G. Goswami, Kamal V. Kanodia

**Affiliations:** Institute of Kidney Diseases and Research Centre–Dr HL Trivedi Institute of Transplantation Sciences, Ahmedabad, India

**Keywords:** Plasmodium vivax, acute kidney injury, hemodialysis, malaria, parasites, India

## Abstract

*Plasmodium vivax* is causing increasingly more cases of severe malaria worldwide. Among 25 cases in India during 2010–2011, associated conditions were renal failure, thrombocytopenia, jaundice, severe anemia, acute respiratory distress syndrome, shock, cerebral malaria, hypoglycemia, and death. Further studies are needed to determine why *P. vivax* malaria is becoming more severe.

India is a major contributor to the worldwide distribution of *Plasmodium vivax* malaria (*1*). Severe and complicated malaria is usually caused by the *P. falciparum* parasite*,* but *P. vivax,* usually considered a benign parasite that causes disease resulting in low case-fatality rates, can also occasionally cause severe disease. Reported severe manifestations of *P. vivax* include cerebral malaria, liver dysfunction, acute kidney injury, severe anemia, acute respiratory distress syndrome, shock, abnormal bleeding, and multiple organ failure (*2**–**10*). The mechanism of *P. vivax*–associated acute kidney injury and its effective management remain unclear. In addition, little information is found in the literature to explain the recent increase in incidence of acute kidney injury and the shift toward multiple complications, specifically in India (*11*). This scarcity of data prompted us to review cases at the Institute of Kidney Diseases and Research Centre–Institute of Transplantation Sciences, Civil Hospital, Ahmedabad, India.

## The Study

**Figure 1:**
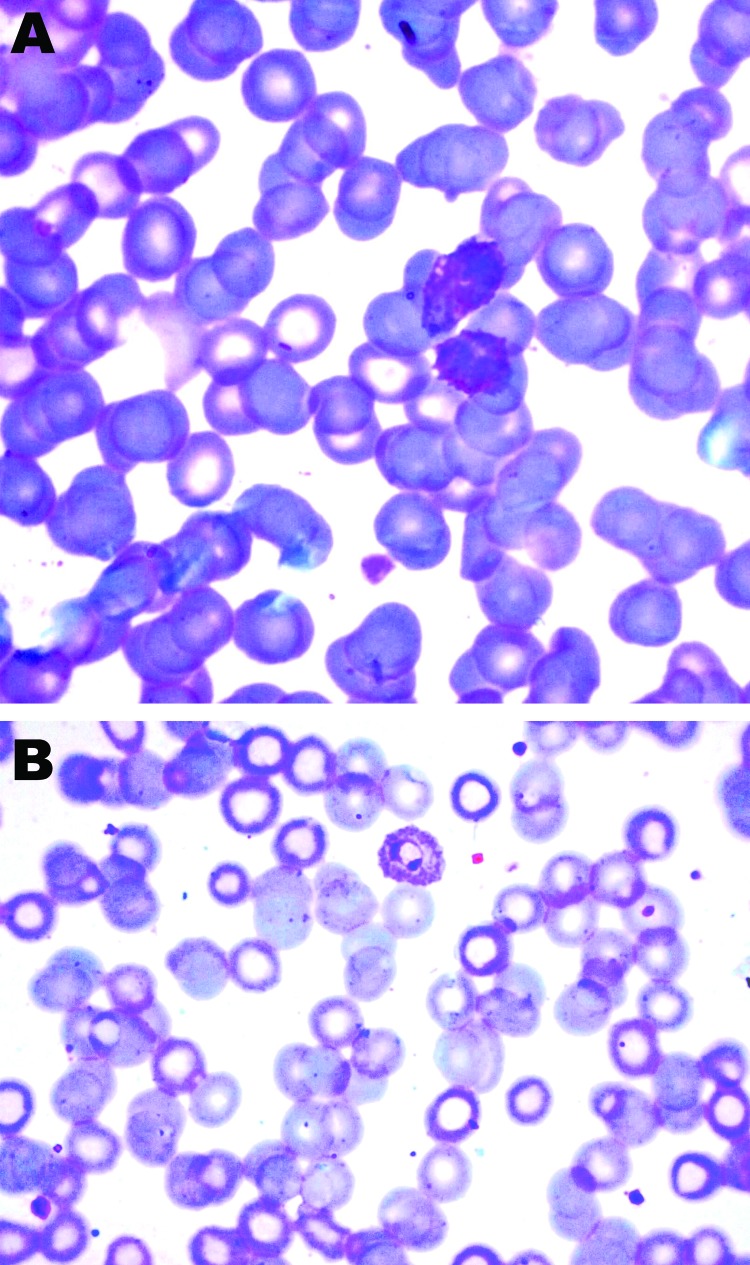
Schizonts (A) and ring form (B) of *Plasmodium vivax*, Ahmedabad, India, 2010–2011. Original magnification ×1,000.

We conducted a prospective study during 2010–2011 to describe clinical characteristics, laboratory parameters, prognostic factors, and outcomes for 25 Civil Hospital patients who required hemodialysis for acute kidney injury associated with *P. vivax* monoinfection. *P. vivax* monoinfection was diagnosed by direct visualization of the parasite in Giemsa-stained peripheral blood films (Figure 1) and rapid diagnostic test results (negative for histidine-rich protein 2 of *P. falciparum* and positive for *P. vivax*–specific lactate dehydrogenase). The severity of illness was assessed by using the Acute Physiology and Chronic Health Evaluation (APACHE II), Sequential Organ Failure Assessment (SOFA), Multiple Organ Dysfunction Score (MODS), and Glasgow Coma Scale. Smear evaluation included reading 200–500 fields with the oil immersion objective lens for >20 minutes by an expert. If the initial smear examination was negative for *P. falciparum*, additional smears were reviewed within 6–24 hours to rule out mixed infection. Patients with other concurrent illness or *P. falciparum* mixed infections were excluded from the study. After a diagnosis was established and treatment was initiated, the parasitologic response was monitored. Other causes of acute kidney injury, fever, and jaundice (i.e., dengue viral infection, leptospirosis, sepsis, typhus, enteric fever, and viral hepatitis and drug reactions) were excluded by history and relevant investigations.

Acute kidney injury was defined as serum creatinine >3 mg/dL and urine output <400 mL/24 hours with normal kidney size according to ultrasonography. The patients were treated with antimalarial drugs (artesunate and doxycycline [n = 25], quinine [n = 2]); fluid replacement and supportive measures were instituted as needed. Intravenous ceftriaxone was given initially until a bacterial infection (e.g., *Salmonella* infection) was excluded. Renal replacement therapy was initiated before overt symptoms and signs of acute kidney injury developed (*10**,**11*). Hemodialysis was initiated for fluid overload, hyperkalemia, clinical evidence of uremia, metabolic acidosis, blood urea nitrogen >100 mg/dL, and creatinine >4–5 mg/dL or rapidly increasing. Intermittent hemodialysis (n = 24) was provided on alternate days through a temporary dialysis catheter, and continuous renal replacement therapy was provided for 1 hemodynamically unstable patient. Renal replacement therapy continued until the patient’s kidney function improved (increase in urine output or progressive decline in creatinine).

Blood transfusion was reserved for patients with hemoglobin <7 g/dL. Exchange transfusion was performed for patients with parasite density >10%, end-stage organ failure complications, and serum bilirubin >25 mg/dL. Renal biopsy was performed when oliguria, heavy proteinuria, or hematuria persisted for >3 weeks.

Of the 202 malaria patients in whom acute kidney injury was treated, 177 (87.7%) cases of total malaria with acute kidney injury were caused by *P. falciparum* and 25 (12.3%) by *P. vivax*. Baseline characteristics of the study patients are shown in Table 1. Comparison of patients who survived or died is shown in Table 2. Initial clinical features were fever (100%), nausea or vomiting (84%), oliguria (60%), abdominal pain or tenderness (40%), jaundice (40%), dyspnea (32%), diarrhea (12%), altered sensorium (4%), and convulsions (4%). The time between onset of symptoms and admission to the hospital for acute kidney injury was 7–15 days (median 11 days). At admission, cultures of blood, sputum, and urine were negative for microorganisms for all patients. The following complications were observed: renal failure (100%), thrombocytopenia (64%), thrombocytopenia <50,000 cells/μL (40%), hyponatremia (52%) (usually asymptomatic), jaundice (bilirubin >3 mg/dL) (40%), severe anemia (hemoglobin <7 gm/dL) (40%), acute respiratory distress syndrome (16%), shock (12%), cerebral malaria (4%), hypoglycemia (4%), and death (12%).

**Table 1:** Baseline values for 25 *Plasmodium vivax* patients with acute kidney injury, Ahmedabad, India, 2010–2011*

Characteristic	Value
Age, y	30.1 ± 12.3
Hypotension not resolving after receipt of fluid, no. (%)	3 (12)
Mechanical ventilation, no. (%)	4 (16)
Lactate dehydrogenase, U/L	1267 ± 462
APACHE 2 score	17.4 ± 3.8 (9–27)
SOFA score	8.4 ± 2.8 (4–14)
MODS score	7.9 ± 2.8 (4–14)
GCS score	14.3 ± 1.14 (12–15)
Hemoglobin, gm/L	8.02 ± 1.6
Hematocrit, %	24.6 ± 4.9
Leukocytes, cells/mm^3^	9,002.8 ± 3,098
Platelets, cells/μL	108,680 ± 91,606
Total bilirubin, mg/dL	5.6 ± 7.6
Direct bilirubin, mg/dL	4.1 ± 6.2
Indirect bilirubin, mg/dL	1.5 ± 2.1
Alanine aminotransferase, U/L	66 ± 45.5
Sodium, mmol/L	130 ± 6.8
Creatinine, mg/dL	7.37 ± 2.6
Blood urea, mg/dL	140.4 ± 59
No. hemodialysis sessions	5.1 ± 3.5

*Values are mean ± SD (range) except as indicated. APACHE, Acute Physiology and Chronic Health Evaluation; SOFA, Sequential Organ Failure Assessment; MODS, Multiple Organ Dysfunction Score; GCS, Glasgow Coma Scale.

**Table 2:** Comparison of 25 *Plasmodium vivax* patients with acute kidney injury, Ahmedabad, India, 2010–2011*

Characteristic	Survived, n = 22	Died, n = 3	p value
Age, y	27.7 ± 9.9	47.3 ± 17.5	0.058
Hypotension not resolving after receipt of fluid, no. (%)	0	3 (100)	0.0001
Mechanical ventilation, no. (%)	1 (4.5)	3 (100)	0.0001
Lactate dehydrogenase, U/L	1126.3 ± 243.2	2300 ± 360.5	0.001
APACHE 2 score	16.4 ± 2.61 (9–23)	25.3 ± 1.52 (24–27)	0.001
SOFA score	7.73 ± 2.3 (4–11)	13.3 ± 1.15 (12–14)	0.001
MODS score	7.23 ± 2.26 (4–10)	13 ± 1.0 (12–14)	0.001
GCS score	14.5 ± 1.01 (12–15)	12.3 ± 0.57 (12–13)	0.013
Hemoglobin, gm/L	8.0 ± 1.71	8.13 ± 0.70	0.96
Hematocrit, %	24.5 ± 5.2	25.6 ± 2.3	0.78
Leuckocytes, cells/mm^3^	9,175.9 ± 3,110	7,733.3 ± 3,300	0.49
Platelets, cells/μL	113,409 ± 96,618	74,000 ± 2,5159	0.90
Total bilirubin, mg/dL	6.1 ± 8	2.56 ± 0.75	0.72
Direct bilirubin, mg/dL	4.46 ± 6.58	1.76 ± 0.40	0.60
Indirect bilirubin, mg/dL	1.64 ± 2.23	0.80 ± 0.34	0.90
Alanine aminotransferase, U/L	66.9 ± 47.8	59.3 ± 29.1	0.96
Sodium, mmol/L	130.5 ± 7.17	126.6 ± 2.3	0.23
Creatinine, mg/dL	7.36 ± 2.7	7.5 ± 2.17	0.66
Blood urea, mg/dL	147.7 ± 57.5	87 ± 48.8	0.08
No. hemodialysis sessions	5.3 ± 3.7	3.33 ± 1.15	0.23

*Values are mean ± SD (range) except as indicated. APACHE, Acute Physiology and Chronic Health Evaluation; SOFA ,Sequential Organ Failure Assessment; MODS, Multiple Organ Dysfunction Score; GCS, Glasgow Coma Scale.

Urinalysis showed muddy brown granular casts or epithelial cell casts; trace protein; and absence of dysmorphic red cells, heavy protein, and leukocytes. None of the patients were deficient in glucose 6-phosphate dehydrogenase activity in erythrocytes. The mean APACHE II, SOFA, MODS, and Glasgow Coma scores were higher for patients who died than those who survived (Table 2). SOFA and MODS scores <12 were associated with a low mortality rate (22 patients, 0 deaths), whereas scores >12 indicated a worse outcome (3 patients, 3 deaths). APACHE II score <24 was associated with a low mortality rate (22 patients, 0 deaths), whereas score >24 indicated a worse outcome (3 patients, 3 deaths) (p<0.008).

**Figure 2:**
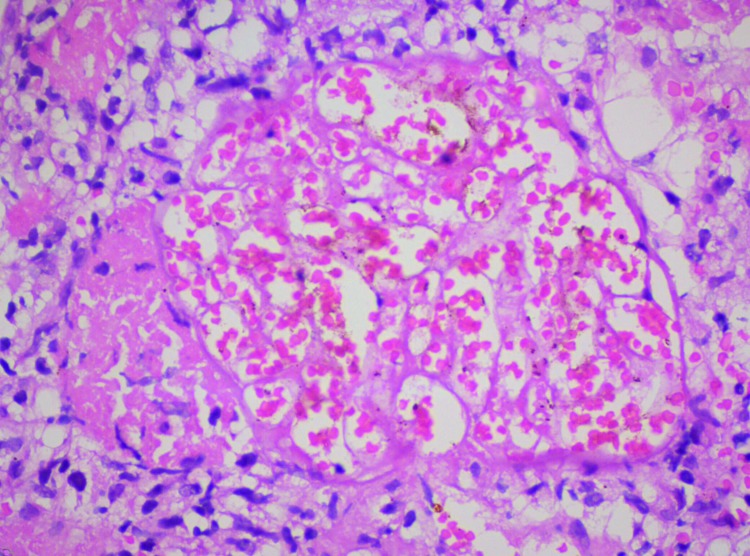
Renal biopsy sample showing patchy cortical necrosis, hematoxylin and eosin staining, Ahmedabad, India, 2010–2011 Original magnification ×400.

Cerebral malaria occurred in 1 patient, who died. Blood component transfusion was given to 7 (28%) patients. Exchange transfusion was given to 1 (4%), who survived. For all patients, initial rehydration failed to lead to renal recovery. Most patients no longer needed dialysis after 2 weeks, and renal function returned in 3 weeks. Renal biopsies, performed for 4 patients, detected patchy cortical necrosis in 3 (Figure 2) and acute tubular necrosis in 1. A total of 19 (76%) patients completely recovered with normal renal function (creatinine <1.4 mg/dL [reference 0.7–1.4 mg/dL]), 3 (12%) did not recover completely (creatinine 1.5–3 mg/dL) and continued to receive conservative treatment, and 3 (12%) died. Factors related to acute kidney injury were heavy parasitemia (48%), hyperbilirubinemia (40%), volume depletion (40%), intravascular hemolysis (100%), and sepsis (12%).

## Conclusions

Our study highlights the possibility that *P. vivax* can cause acute kidney injury. There is no direct pathogenic linkage between *P. vivax* and acute kidney injury. Hence, despite the association, cause-and-effect relationships remain doubtful. However, the following can contribute to acute kidney injury: heavy parasitemia, volume depletion, hyperbilirubinemia, intravascular hemolysis, renal ischemia, sepsis, disseminated intravascular coagulation, cytoadherence to endothelial cells, and microvascular sequestration (*2**,**10**,**11**,**12**,**13*). Bilirubin and hemoglobin were useful for predicting *P. vivax*–induced nephropathy (*9*). Because co-infection with *P. vivax* and *P. falciparum* protects patients from severe malaria, mixed infection is unlikely in patients with severe malaria (*2**,**9**,**14*).

In our study, APACHE II score >24 and SOFA and MODS scores >12 were associated with higher mortality rates. These scores can be used for prognosis for patients with malaria and acute kidney injury and can help us better categorize patients for better management and improved outcomes. One limitation of our study is that PCR confirming *P. vivax* monoinfection was not performed routinely.

Acute cortical necrosis is rare in patients with acute kidney injury from malaria (*15*). The possible pathogenic factors are renal damage through renal hypoperfusion or endothelial injury through release of various circulating substances (intravascular hemolysis and sepsis). *P. vivax* should be suspected in patients with acute kidney injury who have prolonged oligoanuria. *P. vivax* can lead to serious, potentially life-threatening complications, such as acute kidney injury. Further studies are needed to determine why *P. vivax* infections are becoming more severe.
